# transAlign: using amino acids to facilitate the multiple alignment of protein-coding DNA sequences

**DOI:** 10.1186/1471-2105-6-156

**Published:** 2005-06-22

**Authors:** Olaf RP Bininda-Emonds

**Affiliations:** 1 Lehrstuhl für Tierzucht, Technical University of Munich, Hochfeldweg 1, 85354 Freising-Weihenstephan, Germany

## Abstract

**Background:**

Alignments of homologous DNA sequences are crucial for comparative genomics and phylogenetic analysis. However, multiple alignment represents a computationally difficult problem. For protein-coding DNA sequences, it is more advantageous in terms of both speed and accuracy to align the amino-acid sequences specified by the DNA sequences rather than the DNA sequences themselves. Many implementations making use of this concept of "translated alignments" are incomplete in the sense that they require the user to manually translate the DNA sequences and to perform the amino-acid alignment. As such, they are not well suited to large-scale automated alignments of large and/or numerous DNA data sets.

**Results:**

transAlign is an open-source Perl script that aligns protein-coding DNA sequences via their amino-acid translations to take advantage of the superior multiple-alignment capabilities and speed of an amino-acid alignment. It operates by translating each DNA sequence into its corresponding amino-acid sequence, passing the entire matrix to ClustalW for alignment, and then back-translating the resulting amino-acid alignment to derive the aligned DNA sequences. In the translation step, transAlign determines the optimal orientation and reading frame for each DNA sequence according to the desired genetic code. It also checks for apparent frame shifts in the DNA sequences and can handle frame-shifted sequences in one of three ways (delete, align as amino acids regardless, or profile align as DNA). As a set of comparative benchmarks derived from six protein-coding genes for mammals shows, the strategy implemented in transAlign always improves the speed and usually the apparent accuracy of the alignment of protein-coding DNA sequences.

**Conclusion:**

transAlign represents one of few full and cross-platform implementations of the concept of translated alignments. Both the advantages accruing from performing a translated alignment and the suite of user-definable options available in the program mean that transAlign is ideally suited for large-scale automated alignments of very large and/or very numerous protein-coding DNA data sets. However, the good performance offered by the program also translates to the alignment of any set of protein-coding sequences. transAlign, including the source code, is freely available at http://www.tierzucht.tum.de/Bininda-Emonds/ (under "Programs").

## Background

Alignments of homologous DNA sequences are crucial for comparative genomics and phylogenetic analysis [1]. The most accurate multiple alignment tool arguably remains the human eye. However, the increasing amount of sequence data and the increasing scope of projects using these data mean that an automated alignment procedure is often necessary at some point to achieve the final alignment.

For protein-coding DNA sequences, alignments obtained from the amino-acid residues specified by the DNA sequences will often be superior to those obtained directly from the DNA for several reasons (see also [2]). First and foremost, aligning the amino-acid residues preserves the codon structure of the coding sequence, thereby avoiding the introduction of any frame shifts through the alignment process. Second, because amino acids are more conserved evolutionarily than DNA, and possibly because the amino-acid alphabet is larger than the DNA one and therefore less likely to become saturated with convergent substitutions over longer timeframes, it is often easier to align amino-acid sequences between more distantly related organisms. Third, unlike for nucleotide data, the transition matrices that exist for amino-acid data (e.g., BLOSUM [3], GONNET [4], or PAM [5]) are empirically derived and thus perhaps more "biologically realistic". The many different possible models of nucleotide evolution (see [6]) and the fact that different genes evolve according to different models makes the likelihood of obtaining an equivalent, global nucleotide transition matrix small. Finally, because the translated amino-acid sequence is one-third as long as the original DNA sequence, the alignment procedure will be faster. Based purely on the differences in sequence length, the speedup would be on the order of a factor of nine, given that the Smith-Waterman [7] algorithm for the pairwise alignment of sequences that underlies many multiple-alignment programs runs in *O*(*n*^2^), where *n *= length of the sequence (i.e., is proportional to the product of the lengths of the sequences). However, other considerations, including the speed of the different scoring routines that could be implemented for DNA versus amino-acid data or the memory usage and general implementation of the system, will also be important determinants of the final relative speed increase.

One limitation to aligning amino-acid residues is that the redundancy of the genetic code, whereby up to six sets of nucleotide triplets can specify the same amino acid, means that it is not possible to back-translate an amino-acid sequence without recourse to the corresponding DNA sequence. Numerous programs exist to back-translate aligned amino-acid sequences – for example, the standalone version of RevTrans [2] and mrtrans [8] – but most require both the aligned sequences and the corresponding, unaligned DNA sequences as input. As such, the investigator must determine the proper translation frame for each sequence and perform the amino-acid alignment beforehand, which does not lend itself to the automated alignment of large numbers of DNA sequences.

The server version of RevTrans [9] goes a step further by optionally taking DNA sequences as input, virtually translating them into their respective amino-acid sequences, aligning the latter using DIALIGN2 [10], and then back-translating to achieve the DNA alignment. Altogether, this strategy makes use of the superior and faster alignments produced by amino-acid data, while retaining the greater information content of the DNA sequences for future analyses. Similar functionality is also built directly into DIALIGN2. However, the RevTrans server is limited to only 75 DNA sequences and does not perform any preprocessing of them. As such, is not well-suited to the automated alignment of large numbers of sequences. Both RevTrans and DIALIGN2 also make use of only the BLOSUM transition matrix. LAGAN and Multi-LAGAN [11] also offer the possibility of "translated alignments" (via the translated anchoring option), but both programs are geared more toward the alignment of long, genomic sequences.

Building on these solutions, transAlign (for *trans*lated *align*ments) provides the same basic functionality as the RevTrans server, but with no constraints on the number of input sequences (beyond the memory of the user's computer) and a wider selection of amino-acid transition matrices. More importantly, transAlign also offers a suite of user-defined options (described below) for manipulating either the raw sequence data or the aligned sequences. The most of important of these options relate to DNA sequences that do not translate into "clean" amino-acid sequences and thus could impact negatively on the amino-acid alignment. Together with it being a standalone program, these features make transAlign suitable for both individual data sets and as part of a pipeline for the automated alignment of large numbers of sequences downloaded directly from any of the sequence databases.

## Implementation

transAlign can automatically read DNA sequences in any of four formats: fasta, nexus [12], classic [13] or "extended" [14] PHYLIP, and Se-Al [15]. It can also write the final alignment in any of these same formats. (Conversion to or from additional formats can be accomplished through other programs such as readSeq [16] or sreformat, part of the HMMER package [17].) Some basic filtering of the DNA sequences is also implemented, including the stripping of gaps (either all gaps or only those flanking a sequence) and deleting sequences with more than a user-defined percentage of ambiguous nucleotides (i.e., Ns).

After initial processing of the DNA sequences, transAlign will determine the optimal translation for each sequence according to any of the genetic codes listed by the NCBI [18]. It is also possible for Se-Al formatted data to have different genetic codes specified for each sequence. As far as possible, transAlign translates codons containing ambiguous nucleotides (but not explicit gaps). The optimal translation is held to be that yielding the fewest stop codons excluding the terminal codon. By default, only the three reading frames for the input orientation are examined; however, it is possible to examine the complemented, reversed, and reverse-complemented orientations as well. For equally optimal orientations, transAlign favours the one perturbing the original DNA sequence the least: in order, 1) the orientation as input, followed by the second and third reading frames in that orientation, and then the respective reading frames in each of the 2) complementary, 3) reverse, and 4) reverse-complementary orientations.

transAlign then passes the translated sequences to ClustalW [19,20] for alignment (according to any of the BLOSUM, GONNET or PAM protein weighting matrices) and back-translates the resulting aligned residue sequences into aligned DNA sequences. ClustalW was chosen because it is perhaps the best known and most widely used multiple-alignment program. It also offers the largest choice of amino-acid transition matrices (BLOSUM, GONNET, and PAM) and the ability to do profile alignments (see below). However, slight modifications to the transAlign code would allow the use any suitable multiple-alignment program that accepts protein sequence data as input (e.g., DIALIGN2 with its Clustal-like output in particular). Regardless of the alignment program used, it is expected that increases in both speed and accuracy compared to aligning the sequences as DNA would still occur given the many advantages for aligning protein-coding DNA sequences as amino acids (see above).

An option is also provided to automatically delete any poorly aligning sequences as determined by the initial pairwise alignments performed by ClustalW. This feature is intended largely to remove problematic sequences from alignment pipelines, where it is difficult to (manually) improve the global alignment afterwards. For each sequence, the mean of its pairwise alignment scores is compared to that between all the remaining sequences according to a one-tailed two-sample t-test corrected for multiple comparisons. As such, the procedure is most effective at identifying isolated problematic sequences, which might derive from the inclusion of a potential paralog or simply a misidentified sequence. Families of such sequences (e.g., if the data set contains numerous copies of each of the paralogs from a gene family) are less likely to be detected.

Because ClustalW ignores ambiguous amino acids and stop codons (neither being present in the amino-acid transition matrices), transAlign translates them initially as gaps to permit back-translation. This procedure is unproblematic unless the ambiguous residue or stop codon is adjacent to a gap inferred by the alignment procedure, where it could be placed at either the start or end of the gap. For ambiguous residues arising from incomplete codons, transAlign determines the more optimal of the two placements based on the concordance of the missing nucleotide(s) with the gap. However, all such instances should still be examined and, if necessary, corrected for on an individual basis during the manual inspection that follows any automated alignment procedure.

Obviously, the use of transAlign is restricted to coding DNA sequences only and should not be used for non-coding DNA, whether for genes such as 18S rDNA (= *MTRNR2*; [21]); flanking UTR, regulatory, or intronic regions of genes; or microsatellite sequences. The procedure is also adversely affected by frame shifts (e.g., from sequencing errors). Therefore, transAlign will minimally issue a warning for each sequence that contains more than a user-specified threshold of stop codons (excluding the terminal codon) in the optimal orientation. This threshold can either be an absolute number of stop codons (default) or a percentage of stop codons in the remaining sequence after the first stop codon is encountered. Although this procedure is generally robust, it is less likely to detect frame shifts that occur near either end of a given sequence because of the reduced probability of an erroneous stop codon arising in the few remaining resides.

**Figure 1:**
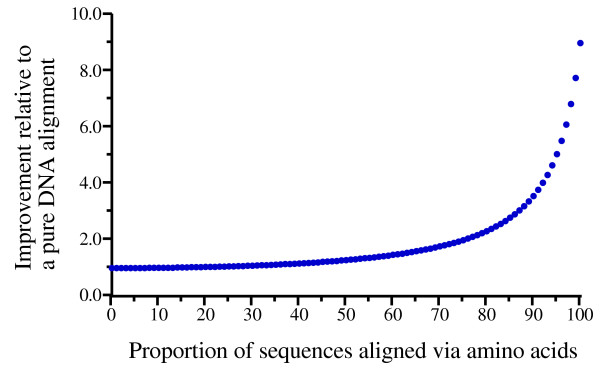
Theoretical gain in speed from performing a translated alignment. The figure reveals there is always a performance advantage in aligning any given proportion of the protein-coding DNA sequences in a data set via their amino-acid translations with the remaining DNA sequences subsequently profile-aligned to them. The curve as shown is based on the assumption that the translated alignment is 9x faster, on average, than the respective DNA alignment; other values produce nearly identical curves of different scales.

Three global solutions for any frame-shifted sequences are implemented in transAlign: 1) deletion, 2) alignment using the translated sequences regardless (with the associated errors), or 3) subsequent profile alignment as DNA to the aligned set of non-frame-shifted sequences (default). The latter option is the slowest of the three, but allows all sequences to be aligned as robustly as possible. Moreover, even a partial profile alignment will always be faster than aligning all sequences as DNA (Figure 1), regardless of the actual speedup inherent to aligning the shorter amino-acid sequences. However, performance will drop off quickly as the proportion of frame-shifted sequences in the data set increases. For instance, assuming a speedup of 9x for aligning amino acids compared to DNA (which, as mentioned, is the value expected based only on length considerations), the overall time saving will only be about 2x if frame-shifted sequences comprise 25% of all sequences (see Figure 1). Finally, to facilitate the manual inspection of the dataset, transAlign will also attempt to infer putative locations for frame-shifting indels based on a comparison of gaps between the amino-acid aligned and DNA profile-aligned sequences.

As mentioned above, transAlign will output the aligned DNA sequences in any or all of fasta, nexus, (classic or extended) PHYLIP, or Se-Al formats. By default, the sequences are output in alphabetical order according to their name. However, it is also possible to output them to match their order in the original input file or as they were output from the ClustalW alignment. The latter option is particularly useful at identifying "families" of similar sequences or those sequences that were profile-aligned to facilitate any manual correction of the global alignment.

transAlign is written in Perl and is open source. It will run on any operating system with a Perl interpreter and is command-line driven. However, it also features a user-interactive mode where the user is prompted to set all the relevant variables. It requires that a remotely-callable version of ClustalW is present either in the global path or in a user-specified one. Again, however, slight modifications to the code would allow the use any suitable multiple-alignment program.

## Results and discussion

To test the potential performance advantages offered by a translated alignment of protein-coding DNA sequences, six mammalian coding genes were each aligned either directly using ClustalW (default parameters) or via their amino-acid translations using transAlign (genetic code specified, otherwise default parameters). All alignments used ClustalW v1.83 on an 800-MHz dual-processor Macintosh G4 running OS 10.3.5. The qualities of the respective alignments were judged relative to a manual alignment of the same data set, each of which was completed for other purposes prior to transAlign being written. As such, the manual alignments represent reasonable, independent reference points. Quality was quantified by calculating the opposite of the Hamming distance (i.e., matching nucleotides score +1; mismatches score +0) between the same sequence in the test alignment and the manually produced one. These values were then averaged for each data set to essentially reveal how many nucleotides, on average, were correctly aligned.

The benchmark data (Table 1) show that transAlign indeed delivers alignments of often superior quality compared to a DNA alignment of the same data set, but always with a significant savings in time. In particular, the speedup was usually 7x or greater, and approximately the theoretical 9x for the three cases where a profile alignment was not performed. The only exception was for *RBP3*, where the many sequences that were identified as having possible frame shifts (61 of the 484 in the data set) necessitated an extensive DNA profile alignment. Even so, the overall speedup for this data set remained greater than 3x, in line with theoretical expectations based on the proportion of frame-shifted sequences (see Figure 1). In all cases, accuracy was either comparable to or significantly exceeded that of a DNA alignment. For *MTCYB*, the largest data set examined, the improvement in the alignment score was substantial (~2x), with the translated alignment requiring only 1.6 days as compared to over two weeks for the DNA sequence data.

It should be kept in mind that these benchmarks serve largely to point out the performance advantages inherent to performing a translated alignment. Other multiple-alignment programs that are faster than ClustalW do exist. But, the same advantages would also apply to these programs, such that alignments for the benchmark data sets could be obtained in even less time.

## Conclusion

The principle underlying transAlign – that of aligning protein-coding DNA via its amino-acid translation – is not novel, having been suggested at least since the initial release of mrtrans (circa 1993). However, together with LAGAN, Multi-LAGAN, DIALIGN2, and the RevTrans server, transAlign represents one of the few complete implementations of the principle, with most of the remaining methods requiring the user to manually translate the DNA sequences and perform the amino-acid alignment. However, transAlign, in addition to being cross-platform, also includes a diverse suite of user-definable options relating to the processing of the DNA sequence data, its alignment as amino-acid data, and subsequent back-translation into aligned DNA data. In particular, transAlign uniquely offers different options to process sequences that do not translate into clean amino-acid sequences and, as such, may disrupt the alignment procedure. All these options mean that transAlign is well suited for the large-scale automated alignment of very large and/or very numerous data sets. As the benchmark studies show, the use of translated alignments provides alignments of at least comparable and often improved quality compared to a DNA alignment and always with a significant savings in time.

## Availability and requirements

Project name: transAlign

Project home page: http://www.tierzucht.tum.de/Bininda-Emonds/ (under "Programs")

Operating system: Unix-based systems including OS X and Linux; DOS

Programming language: Perl; no additional modules required

Other requirements: ClustalW or, with suitable modifications to the source code, most other multiple-alignment programs

License: None; open-source

Any restrictions to use by non-academics: None

## Figures and Tables

**Table 1:** Benchmark data for the comparative performance of a translated alignment. Six mammalian protein-coding genes were aligned either as DNA (using ClustalW; default parameters) or via their translations as amino acids (using transAlign; genetic code specified, otherwise default parameters). All analyses used ClustalW v1.83 on an 800-MHz dual-processor Macintosh G4 running OS 10.3.5. The alignment score is taken relative to the corresponding sequence from a manually aligned data set and is the opposite of the Hamming distance (i.e., matching bases score +1, mismatches score +0). The alignment score was calculated for each individual sequence and then averaged over all sequences in each data set. Gene symbols follow the HUGO Gene Nomenclature Committee (HGNC; [21]).

					Amino-acid alignment				
					
			DNA alignment		Time (sec)				
				
Data set	No. of sequences	Unaligned sequence length	Alignment time (sec)	Average alignment score	Amino-acid alignment	DNA profile alignment	transAlign processing	Total	Average alignment score
*BDNF*	100	256-768	475	579.28	52	14	0	66	774.61
*MTCYB*	2484	388-1200	1216963	437.54	127309	13823	34	141166	860.75
*RAG1*	128	543-3141	2804	2346.46	307	n/a	3	310	2345.13
*RAG2*	196	326-1584	6492	1583.85	733	n/a	3	736	1583.95
*RBP3*	484	627-1292	45122	598.26	4004	10636	9	14649	579.71
*VWF*	182	711-1310	8384	862.06	921	n/a	4	925	1002.16
